# Tc-HDP quantitative SPECT/CT in transthyretin cardiac amyloid and the development of a reference interval for myocardial uptake in the non-affected population

**DOI:** 10.1186/s41824-018-0035-1

**Published:** 2018-08-22

**Authors:** Stuart C. Ramsay, Karen Lindsay, William Fong, Shaun Patford, John Younger, John Atherton

**Affiliations:** 1 Department of Nuclear Medicine and Specialised PET Service, Ned Hanlon Building, Royal Brisbane and Women’s Hospital (RBWH), Herston, QLD 4029 Australia; 2 School of Medicine, James Cook University, Douglas, QLD 4811 Australia; 3 Department of Cardiology RBWH, Herston, QLD 4029 Australia; 4 School of Clinical Medicine, Faculty of Medicine, University of Queensland, Heston, QLD 4006 Australia

**Keywords:** Cardiac amyloid, ATTR, Quantitative SPECT, Tc-HDP

## Abstract

**Background:**

99mTechnetium-HDP (HDP) bone scans differentiate transthyretin (ATTR) cardiac amyloid from other infiltrative myocardial diseases. These scans are not quantitative and are assessed by comparing myocardial uptake to bone. This study examined whether quantitative HDP SPECT/CT can discriminate individuals with cardiac ATTR from the population without this disease.

**Methods:**

HDP thoracic xSPECT/CT QUANT (xQUANT) was performed in 29 patients: ATTR cardiac amyloid (*n* = 6); AL cardiac amyloid (*n* = 1); other infiltrative myocardial disease (*n* = 4); no known infiltrative cardiac disease (*n* = 18). SUVmax measured within volumes of interest for whole heart, ascending aorta blood pool, and specific bones. HDP myocardial uptake calculated as whole heart minus blood pool.

**Results:**

The cardiac ATTR group had greater HDP myocardial uptake than those with no known infiltrative disease (*p* = 0.002). AL and other myocardial diseases had uptake indistinguishable from the group with no known infiltrative cardiac disease. The SUVmaxima were sufficiently similar between individuals without cardiac ATTR that a 99% reference interval for HDP uptake could be calculated, providing an upper limit cut point of SUVmax 1.2. Individuals with cardiac ATTR had SUVmax well above this cut point.

**Conclusion:**

Quantitative SPECT/CT can measure HDP myocardial uptake in individuals with normal hearts and those with cardiac ATTR without recourse to comparison with bone. It enables calculation of a reference interval for HDP myocardial uptake in the population without ATTR cardiac amyloid. Using this reference interval single individuals with cardiac ATTR can be accurately discriminated from the non-affected population. This technique uses a NIST traceable calibration source, potentially allowing development of multicentre clinical decision limits. Its role in disease management warrants further assessment.

## Background

Cardiac amyloidosis is an uncommon restrictive cardiomyopathy associated with deposition of amyloid fibrils in the myocardium. These deposits are almost always of either transthyetin (ATTR) or monoclonal light chain (AL) types, though other subtypes occur. ATTR cardiac amyloid can be acquired (wild type), or hereditary (associated with transthyretin genetic variants). Typically cardiac amyloid patients present with heart failure, but presentations with syncope, arrhythmias and unexplained increased left ventricular (LV) wall thickness also occur. Diagnosis can be difficult as previously definitive diagnosis depended on myocardial biopsy, which may be contraindicated in unwell, often elderly patients with relatively poor prognosis as is frequently seen with wild type ATTR (Gillmore et al., 2016). Surrogate diagnostic techniques may therefore be used in ATTR.

Phosphate based nuclear medicine bone scans can differentiate individuals with ATTR cardiac amyloid from the normal population, as well as distinguishing ATTR from cardiac AL and other infiltrative myocardial disorders associated with increased LV wall thickness (Gillmore et al., 2016; Cappelli et al., 2017; Galat et al., 2015). The specificity of bone scans means that in appropriately selected individuals the diagnosis of ATTR cardiac amyloid can be made without endomyocardial biopsy (Maurer et al., 2017; Habib et al., 2017). Bone scans are also very sensitive in diagnosing ATTR cardiac amyloid, and may identify affected individuals prior to echocardiographic evidence of cardiac involvement (Glaudemans et al., 2014). Although a number of different Technetium 99 m (Tc) labelled phosphate based agents have been used for diagnosis of cardiac amyloid, including pyrophosphate (PYP) and the chemically related bisphosphonates 3,3-diphosphono-1,2-propanodicarboxylic acid (DPD) and hydroxymethylene diphosphonate (HDP), their diagnostic accuracy appears equivalent (Gillmore et al., 2016; Andrikopoulou & Bhambhvani, 2017).

Because standard bone scans are not quantitative, when examining for the presence of cardiac ATTR images are assessed using relative indices with heart uptake being compared to bone uptake. The most widely used index is Perugini grade which involves a visual assessment of cardiac uptake compared to bone and soft tissue, usually on planar imaging (Perugini et al., 2005). This method accurately identifies ATTR cardiac amyloid (Gillmore et al., 2016; Perugini et al., 2005; Hutt et al., 2017), but has some limitations, for example in known cardiac ATTR the Perugini grade has not proven useful in differentiating subgroups with different prognosis (Hutt et al., 2017). Although this may be a weakness inherent in the bone scan technique, it might also be because of the limitations of subjective assessment using a 4 point discrete variable, or because it conflates myocardial uptake with bone uptake. The non-quantitative nature of standard bone scans also makes them potentially less useful in assessing disease progression or treatment response. Absolute quantitation of myocardial uptake of radiopharmaceutical might help to overcome these shortcomings.

Quantitative SPECT/CT has recently become commercially available, though its clinical adoption has been limited by a number of technical issues (Bailey and Willowson, 2014; Armstrong and Hoffmann, 2016; Vija, 2017). Ultimately the combination of acquisition and reconstruction parameters that produces interpretable images and clinically relevant quantitative measurements must be determined by undertaking clinical studies and assessing whether the information obtained is useful. The current study examines whether quantitative SPECT/CT HDP bone scans can discriminate individuals with ATTR cardiac amyloid from those with no history of infiltrative myocardial disease, and those with other forms of infiltrative myocardial disease associated with increased LV wall thickness including AL cardiac amyloid. In particular we investigated the concept of establishing a reference interval for HDP myocardial uptake for the population without cardiac ATTR against which a single potentially affected individual can be compared.

## Methods

### Study population

Patients were included based on their attendance at the Department of Nuclear Medicine at the Royal Brisbane and Women’s Hospital (RBWH) for a clinically indicated bone scan requiring SPECT/CT of the thorax between January 2017 and January 2018. Appropriate ethical approval was obtained according to the RBWH human research ethics committee protocols as a clinical audit, and the need for individual written informed consent was waived.

### Bone scans

800 MBq Tc-HDP was injected intravenously. xSPECT/CT QUANT (Siemens Symbia Intevo) of the thorax was performed approximately 3 h (196+/− 24 min) after injection, followed by whole body planar imaging.

Planar whole body images were graded utilising a previously reported slight modification of the Perugini grading system (Perugini et al., 2005; Hutt et al., 2017). The xSPECT images were reconstructed using standard vendor software incorporating appropriate CT based attenuation correction, dual energy scatter correction and OSCGM reconstruction using 8 subsets, 4 iterations, a 10 mm smoothing filter, and a 12 mm Gaussian filter. The resultant images represent a parametric map of activity distribution of Tc-HDP with units of kBq/mL standardised to the time of injection analogous to PET. These values were then corrected for body weight and injected dose to give SUVs.

Image analysis was undertaken using syngo.via (Siemens). The SUV parametric images were displayed in conjunction with the low dose CT for anatomic localisation. Volumes of interest (VOIs) were defined on the low dose CT based on reproducible anatomical landmarks, then projected onto the xSPECT images. These regions included: whole heart; mid septal wall; ascending aorta blood pool at the level of the main pulmonary artery; a single lower thoracic or upper lumbar vertebral body (T12 unless this was abnormal based on a review of the bone scan images, otherwise the nearest normal adjacent vertebral body); body of the sternum (excluding degenerative change or other focal abnormality); and right 4th rib laterally (excluding focal rib abnormalities). The SUVmax of the VOI was obtained.

The anatomical information on the HDP SPECT and low dose non-contrast CT was insufficient to determine precisely within which tissue the SUVmax was located, and in many individuals without ATTR amyloid blood pool was indistinguishable from the myocardium on HDP scan. Hence a correction was applied to the whole heart region using blood pool SUVmax measured in the ascending aorta (corrected whole heart value = whole heart value – blood pool).

In addition bullseye plots of the distribution of HDP in the LV myocardium of the patients with ATTR. The SPECT images of the heart was reoriented to standard cardiac views using Siemens SPECT reconstruction software, then the bullseye plot was constructed using Cedars Cardiac Quantification (Siemens). In those without ATTR there was insufficient myocardial uptake to allow this analysis.

### Late gadolinium enhancement (LGE) Cardiovascular magnetic resonance imaging (CMR)

In 5 of the ATTR patients CMR using a late gadolinium enhancement (LGE) technique was undertaken. In brief 10 min after intravenous injection of gadolinium based extracellular contrast medium CMR (Siemen’s Skyra 3 T) was performed of the heart in diastole using a prospectively triggered segmented inversion recovery spoiled gradient echo sequence with 8 mm short axis slices. These images were reviewed in short axis views and visually compared to the HDP SPECT images. In 2 of the patients a prospectively triggered single shot inversion recovery steady state free precession sequence was also undertaken in a single diastolic interval to acquire 8 mm short axis slices suitable for coregistration with the HDP SPECT images. In these 2 individuals the bone scan images were coregistered to the short axis LGE CMR images using 3D Task Card (Siemens). The SPECT/CT data set was rotated so that its cartesian coordinates matched those of the short axis MRI slices, the location of the cardiac apex of the 2 image sets was matched manually, and the SPECT images were resliced at the same thickness as the MRI acquisition.

### Statistics

Comparison of myocardial uptake between the groups with ATTR amyloid, AL amyloid, other cardiac diseases and those without cardiac disease was undertaken using independent sample t-tests in SPSS / PASW student version 18.0 (IBM, USA).

To examine the practicality of developing a reference interval two different approaches were taken based on the CLSI C28-A3 standards (CLSI, 2008) using MedCalc (MedCalc Statistical Software version 18 (MedCalc Software bvba, Ostend, Belgium; https://www.medcalc.org; 2018)). In the first method the data were assessed for normality, then when this assessment did not exclude a normal distribution, means and standard deviations were obtained and these were used to calculate the 99% upper limit of a reference interval together with the associated 90% confidence interval. Secondly it has been shown that as few as 20 appropriately selected individuals can be used to determine a reference interval provided the data are transformed with a Box Cox transformation, after which a robust statistical method is used (Efron, 1980; CLSI, 2008; Ozarda, 2016; Morrow & Cook, 2011), hence this method was also used to calculate the upper limit of a 99% reference interval and the associated 90% confidence interval.

For further exploration of the potential for developing reference intervals and possible clinical decision limits ROC analysis was undertaken using MedCalc.

## Results

### Study population

Patient characteristics are summarised in Table 1 and Table 2. The patients were divided into 4 subgroups consistent with the EACVI/ESC expert consensus recommendations (Habib et al., 2017) primarily based on the combination of clinical features, presence or absence of abnormal light chains, CMR and/or echocardiograph result, Perugini score on HDP planar scan and biopsy when considered clinically necessary.

- Group 1) ATTR cardiac amyloid: based on clinical features and CMR findings, positive planar bone scan with Perugini score > =2 and absence of abnormal light chains (*n* = 6, 1 female, 5 males, mean age 78).
- Group 2) AL cardiac amyloid: based on the presence of abnormal light chains (in blood), abnormal CMR (reduced LVEF, increased LV wall thickness and LGE), Perugini score 0, positive duodenal biopsy for AL (*n* = 1, male, age 80).
- Group 3) Other cardiac disease: these patients had heart failure with increased LV wall thickness with or without LGE on CMR, but cardiac amyloid was ultimately deemed very unlikely on the basis of no abnormal light chains identified, Perugini score 0 and no family history of ATTR amyloid (*n* = 4, 2 females, 2 males, mean age 70: cardiomyopathy of unexplained cause (*n* = 3); viral myocarditis (n = 1)).
- Group 4) Non cardiac patients: no history of infiltrative myocardial disease (*n* = 18; females 13, males 5, mean age 60, range 44–84). In these patients the bone scans were performed for non-cardiac indications (staging or restaging of malignancy *n* = 12, non-malignant thoracic pain *n* = 6).

**Table 1:** Characteristics of the patients studied subdivided according to final diagnosis (*found to have disseminated malignancy, prognosis considered too poor for further cardiac investigation, **abdominal fat pad biopsy negative for amyloid, haematological diagnosis monoclonal gammopathy of undetermined significance)

Subject	Group	Disease subtype	Sex	Age	Serum proteins	Perugini score	Heart SUV
P1	1	ATTR	m	75	Normal	2	5.41
P2	1	ATTR	m	77	Normal	2	3.40
P3	1	ATTR	m	78	Normal	2	6.35
P4	1	ATTR	m	76	Normal	2	4.90
P5	1	ATTR	f	74	Normal	2	9.43
P6	1	ATTR	m	90	-*	2	4.24
P7	2	AL	m	80	Increased Kappa light chains	0	0.42
P8	3	Cardiomyopathy - unknown cause	m	82	Monoclonal IgG normal bone marrow**	0	0.51
P9	3	Cardiomyopathy - unknown cause	f	50	Normal	0	0.50
P10	3	Cardiomyopathy - unknown cause	m	83	Normal	0	0.23
P11	3	Viral myocarditis	f	65	Normal	0	1.06
P12	4	Back pain	f	84	–	0	0.82
P13	4	Back pain	f	45	–	0	0.70
P14	4	Breast cancer	f	66	–	0	0.20
P15	4	Thyroid cancer with thoracic pain	f	59	–	0	0.69
P16	4	Breast cancer	f	72	–	0	0.63
P17	4	Follow up sacral metastasis	f	44	–	0	0.53
P18	4	Small cell lung cancer	f	61	–	0	0.69
P19	4	Back pain	m	49	–	0	0.51
P20	4	Breast cancer	f	55	–	0	0.47
P21	4	Back pain	m	56	–	0	0.80
P22	4	Breast cancer	f	47	–	0	0.19
P23	4	Breast cancer	f	46	–	0	0.37
P24	4	Prostate cancer	m	70	–	0	0.45
P25	4	Breast cancer	f	56	–	0	0.10
P26	4	Back pain	f	84	–	0	0.50
P27	4	Prostate cancer	m	61	–	0	0.84
P28	4	Prostate cancer	m	70	–	0	0.47
P29	4	Progress TB in spine	f	47	–	0	0.66

**Table 2:** Diagnostic CMR results for the patients with cardiac disease subdivided according to final diagnosis (*CMR not undertaken - see Table 1)

Subject	Group	CMR / Echocardiograph
P1	1	CMR: Increased LVWT basal and mid regions, normal size LV, diffusely abnormal LGE, LVEF 50%
P2	1	CMR: Concentric increased LVWT, dilated LV, diffuse abnormal myocardial LGE LVEF 23%
P3	1	CMR: Concentric increased LVWT, LV upper limit normal size, diffuse abnormal LGE, LVEF 43%
P4	1	CMR: Concentric increased LVWT, LV normal size, diffuse abnormal LGE LV and RV myocardium, LVEF 41%
P5	1	CMR: Concentric increased LVWT, LV normal size, diffuse abnormal LGE LV and RV myocardium, LVEF 60%,
P6	1	Elevated BNP, mildly elevated TnI. Stress MPS negative, LVEF low normal. No echo or CMR*
P7	2	No CMR (GFR < 30 ml/min). Echo: mild septal LVH 1.3 cm, LV dilated. LVEF 32%.
P8	3	CMR: Concentric increased LVWT, dilated LV, LVEF 29%, no significantly abnormal LGE
P9	3	CMR: Concentric increased LVWT, LV normal size, reduced LVEF 42%, no abnormal LGE
P10	3	No CMR. Echo: Concentric LVH, normal LV size, LVEF 35%,
P11	3	CMR: Concentric increased LVWT, normal LV chamber size, LVEF 51%, diffuse abnormal myocardial LGE

### Bone scans

**Fig. 1:**
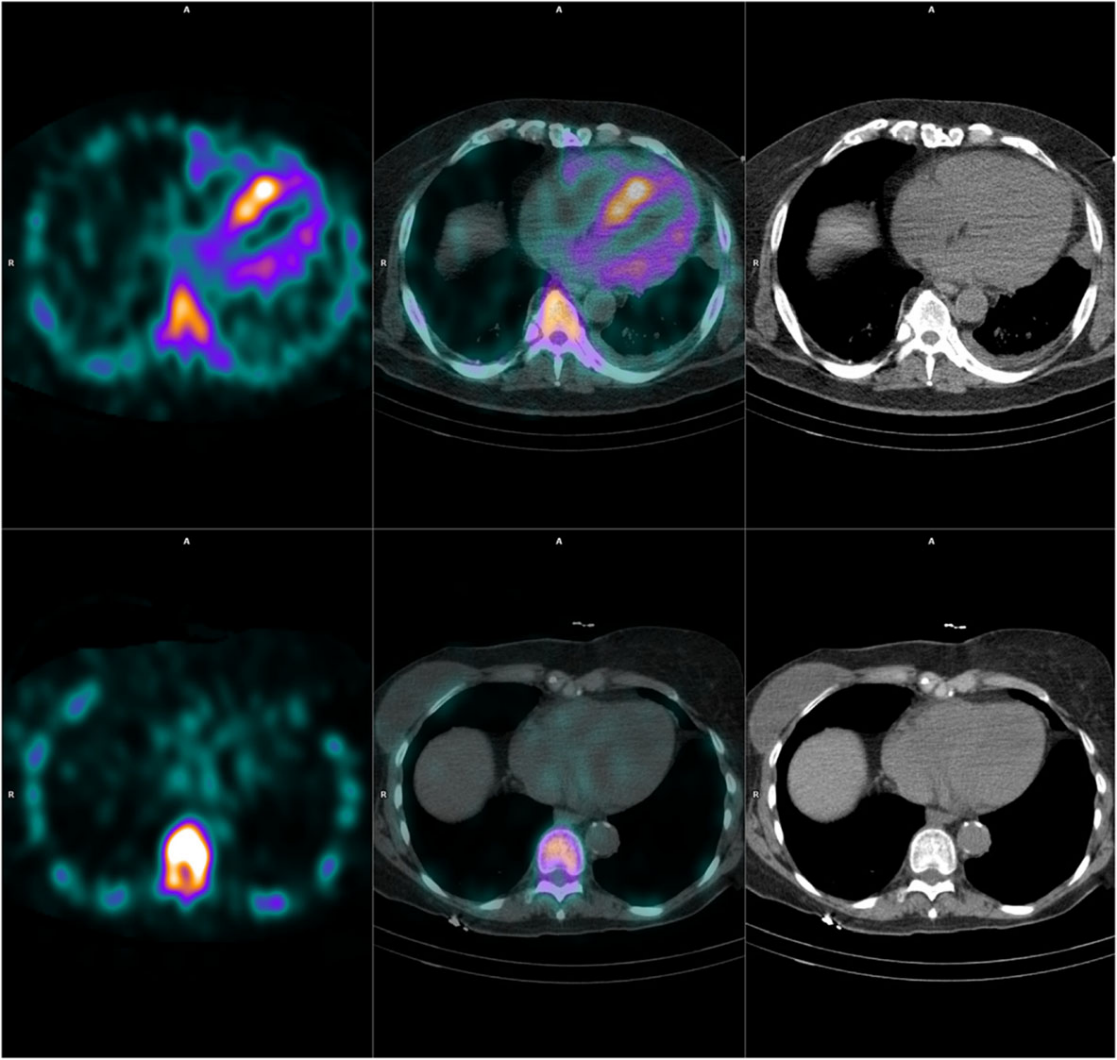
Examples of xSPECT/CT transaxial images through the heart at midventricular level. The upper row is from a patient with ATTR cardiac amyloid, planar imaging in this individual showed Perugini grade 2. The lower row is from a patient with no known heart disease (history of previous right breast cancer)

**Fig. 2:**
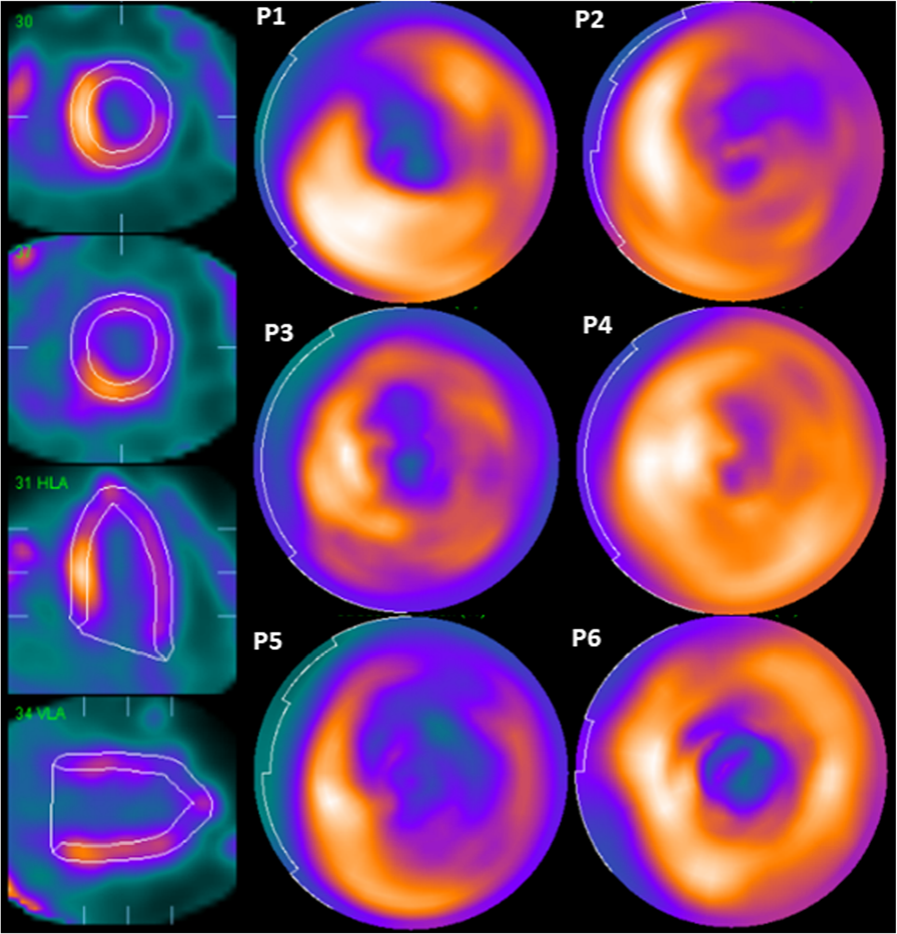
Bullseye plots of HDP distribution in the LV myocardium of each of the patients with ATTR (P1-P6 in Table 1). The myocardial ROIs defined by the program for one patient are shown on the left

**Fig. 3:**
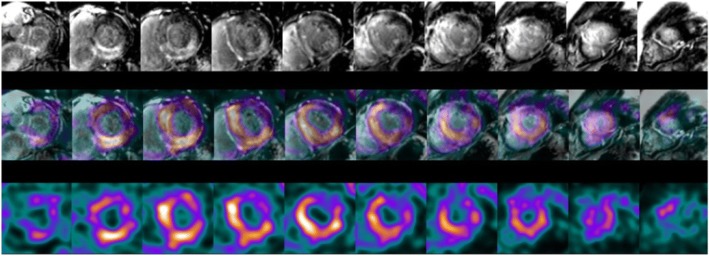
Positive Tc-HDP xSPECT images coregistered to delayed gadolinium enhanced short axis CMR images in a patient with ATTR cardiac amyloid showing the distribution within the myocardium. HDP uptake colocalises to sites of LGE (white regions within myocardium on CMR)

Based on planar images all 6 patients in group 1 (ATTR) had Perugini grade 2, confirming the diagnosis of ATTR cardiac amyloid (Gillmore et al., 2016; Cappelli et al., 2017; Galat et al., 2015; Maurer et al., 2017). Subjects in group 2 (AL), group 3 (other cardiac) and group 4 (non cardiac) all demonstrated Perugini grade 0. xSPECT/CT images in a non-affected individual, and in a characteristic example of ATTR cardiac amyloid, are shown in Fig. 1. As expected from the Perugini scores on planar images, on SPECT HDP uptake in the myocardium was visually more intense in group 1 (ATTR). Uptake was most prominent in the LV myocardium, but was also seen in the RV myocardium and in some cases the atrial walls. Bullseye plots of the 3D distribution of HDP in the left ventricular myocardium in the patients in group 1 (ATTR) are shown in Fig. 2. In those 5 patients in group 1 who had CMR this uptake appears visually to correspond spatially to the distribution of LGE on CMR (Fig. 3).

**Fig. 4:**
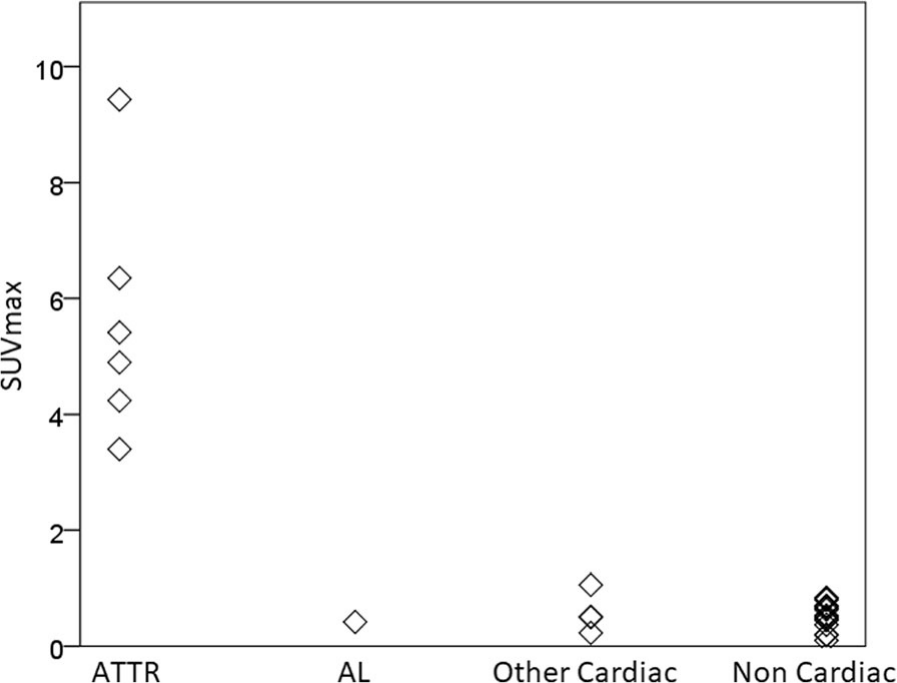
Distribution of whole heart SUVmax measurements corrected for blood pool for subjects according to clinical subgroup

The SUVmaxima obtained from the whole heart ROIs corrected for residual blood pool according to patient subgroups is shown in Fig. 4. The SUVmax within the heart was significantly higher in group 1 (ATTR) than in group 4 (non cardiac with no history of infiltrative myocardial disease) (*p* = 0.002). In groups 2 and 3 (patients with infiltrative myocardial disease / increased LV wall thickness but without ATTR) the SUV max was not significantly different to group 4 (non cardiac): the patient with AL cardiac amyloid showed HDP uptake that was not significantly different to the other individuals without ATTR myocardial infiltration (*p* = 0.62); while group 3 (other cardiac) were similarly not definitely different from group 4 (non cardiac) (*p* = 0.85). Within group 4, there was no significant difference in corrected whole heart uptake according to sex or age (data not shown).

**Fig. 5:**
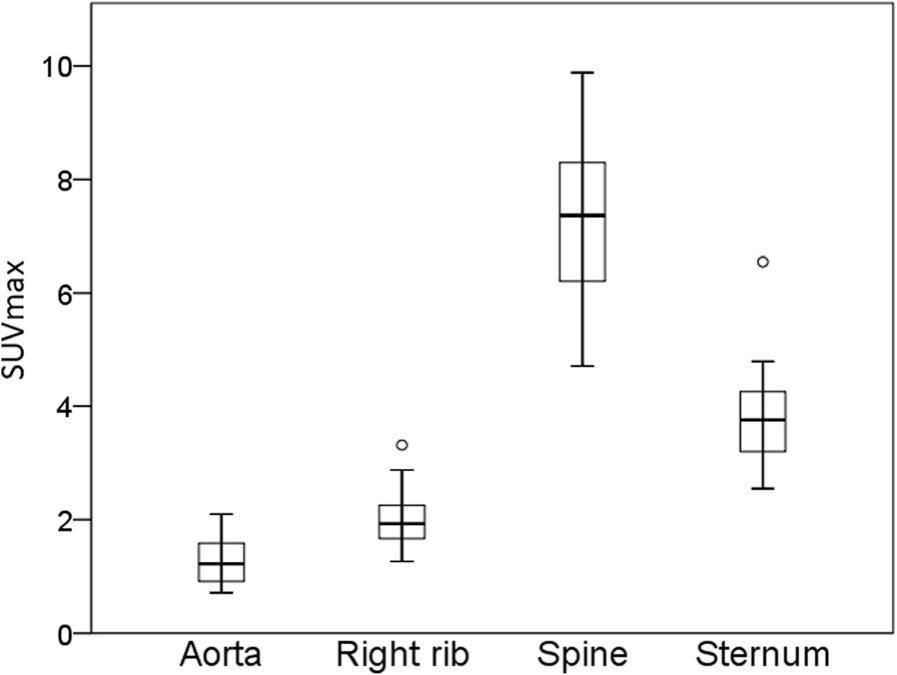
Box and whisker plots for whole heart SUVmax measurements showing medians and interquartile ranges for VOIs other than whole heart. Open circles represent outliers based on Tukey’s test

The distribution of SUVmax for blood pool and selected bone regions for all subjects combined is shown in Fig. 5.

### Reference interval for HDP myocardial uptake

**Fig. 6:**
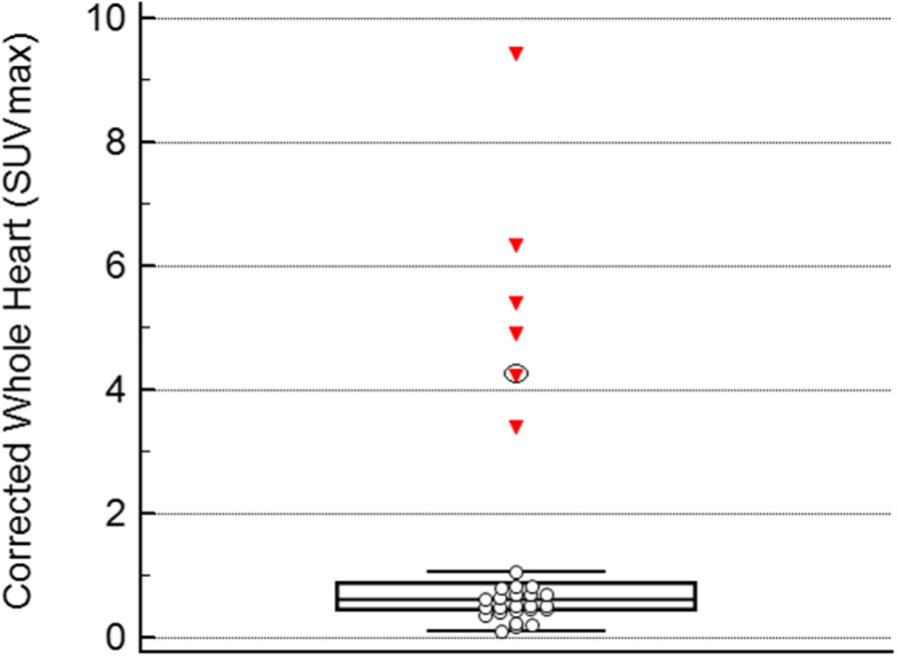
Graphical representation of whole heart SUVmax corrected for blood pool for all subjects considered as a single population, with associated box and whisker plot showing median and interquartile ranges. Open circles represent subjects without ATTR cardiac amyloid. Closed triangles represent statistical outliers using Tukey’s test, and these are the individuals with ATTR cardiac amyloid. Triangle with black ring represents the incidentally identified cardiac ATTR patient with metastatic prostate cancer

**Fig. 7:**
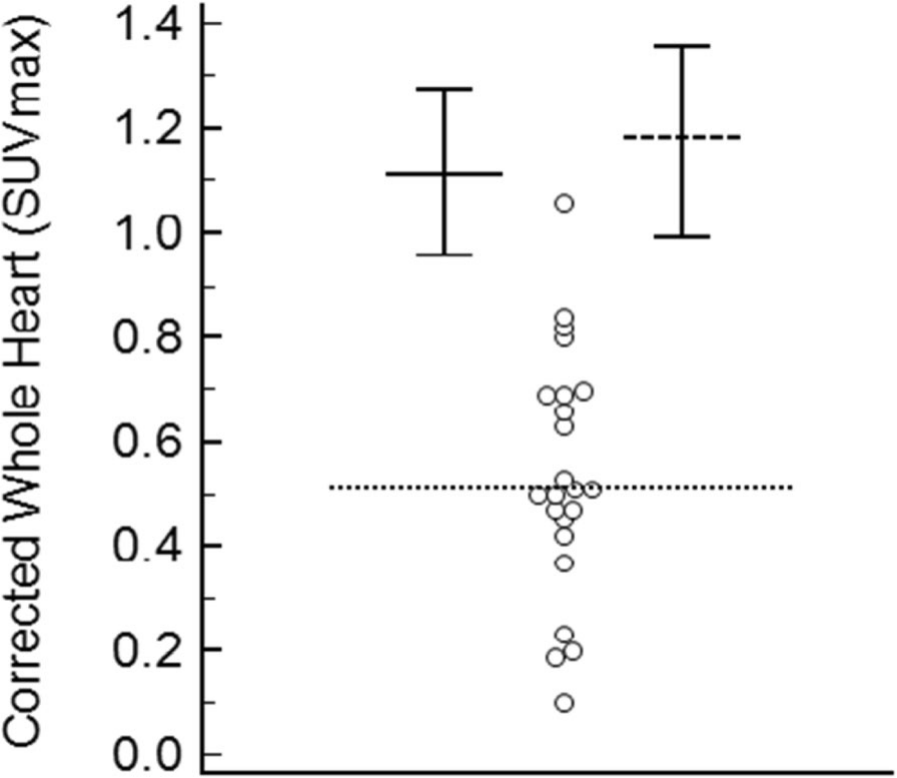
Distribution of corrected whole heart SUVmax for individuals without ATTR cardiac amyloid with the upper limits of 99% reference levels and their associated 90% confidence intervals marked. The dashed horizontal line represents the reference level determined by the robust method, the solid line the reference level determined assuming a normal distribution, and the dotted line is the mean

The subjects appeared to consist of 2 distinct subgroups; those in groups 2, 3 and 4 (without cardiac ATTR) have SUVmaxima that cluster at the low end of the whole heart measurements, while those in group 4 (ATTR) appear to represent outliers relative to this other group (Fig. 6). These outliers were excluded as per CLSI C28-A3 guidelines (CLSI, 2008) leaving 23 individuals sampled from the population with “cardiac ATTR not present”, sufficient for calculation of reference intervals using the robust method (Efron, 1980; CLSI, 2008; Morrow and Cook, 2011; Ozarda, 2016). The distribution of the myocardial uptake for these non-ATTR patients, with appropriately calculated 99% reference intervals with the 90% confidence intervals superimposed is shown in Fig. 7. For the normal distribution method the upper limit was an SUV max of 1.13 (90% confidence interval 0.96–1.29), while the robust method produced an upper limit of 1.18 (90% confidence interval 1.01–1.38). Encouragingly these reference interval upper limits are very similar for the 2 different methods.

Further in regards to determination of clinical decision limits ROC analysis gave 100% sensitivity and specificity at a cut point of 1.06.

Given the relatively small number of subjects and the similarity in cut points determined from the various analysis methods, it was ultimately decided that use of the reference interval determined by the robust method was most appropriate as per the CLSI C28-A3 standard guidelines (CLSI, 2008), with a decision made to round this number up to one decimal place consistent with standard clinical practice for reporting SUVmax in our department. Hence an upper limit cut point for “myocardial HDP uptake in the population not affected by cardiac ATTR” of SUVmax 1.2 was determined. The SUV max of each of the ATTR patients is well above this level (3.40, 4.24, 4.90, 5.41, 6.35, 9.43).

## Discussion

This study shows that quantitative SPECT/CT measurement of HDP myocardial uptake using xQUANT produces meaningful and clinical relevant results. These measurements allow clear demarcation between patients with ATTR cardiac amyloid and those in whom this diagnosis is deemed very unlikely. Unlike previously reported bone scan techniques it does this without any recourse to the degree of uptake in bone. Furthermore, in individuals without cardiac ATTR xQUANT measurements of HDP myocardial uptake were sufficiently robust that a 99% reference interval could be developed for this population using standard CLSI C28-A3c guidelines, despite the relatively small number of individuals studied. The resultant cut point (SUVmax 1.2) enabled accurate discrimination of each individual patient with ATTR cardiac amyloid from the population without cardiac ATTR. An additional advantage of xQUANT is that it uses a National Institute of Standards and Technology (NIST) traceable source for calibration, and this carries with it the potential for using this technique to develop multicentre reference intervals and diagnostic limits.

The relative similarity between individuals for cardiac SUVs also held true for blood pool measurements and bone uptake. The SUVmax values for uptake of HDP in vertebral bodies were similar to those found for Tc-DPD and Tc-MDP by other authors using slightly different quantitative SPECT techniques (Cachovan et al., 2013; Kaneta et al., 2016).

The patient with proven AL cardiac amyloid had myocardial uptake indistinguishable from those with normal hearts, and from those with cardiac infiltration from causes other than ATTR. However the numbers are too small to draw firm conclusions about HDP uptake in AL related cardiac disease. It has been reported that there can be low grade uptake in AL cardiac amyloid (Gillmore et al., 2016; Cappelli et al., 2017; Galat et al., 2015), but those studies used planar imaging rather than SPECT, the degree of residual blood pool was not taken into account, and no attenuation correction is possible on planar images. In the current study we undertook attenuation correction and measured and corrected for blood pool activity, on the basis that this was a potential cause of false positive studies.

It is also worth noting that a continuous variable characterising HDP myocardial uptake without comparison to bone carries with it the potential to monitor changes in cardiac uptake over time in individuals with cardiac ATTR, which would be important in assessing treatment response or measuring disease progression, and potentially may allow a more flexible classification of patients into prognostic subgroups.

The study has a number of weaknesses. Firstly the number of subjects is relatively small. However we used the well established laboratory medicine guidelines CLSI C28-A3c (CLSI, 2008) for establishment of reference intervals appropriate for the number of subjects studied. A number of different analysis techniques were used all of which produced very similar results. In addition the magnitude of the measured differences in HDP myocardial uptake between individuals with ATTR cardiac amyloid and the group with no infiltrative heart disease confirms that this technique for measuring myocardial uptake of HDP is clinically relevant.

Partly because of patient selection the study does not adequately address whether some AL cardiac amyloid patients have low grade HDP uptake. This is because this study was designed around the current clinical practice in our institution, with only selected cardiac patients being imaged with HDP bone scans to confirm or exclude ATTR amyloid. Hence patients with proven AL cardiac amyloid, or with a very high pretest clinical likelihood of AL cardiac amyloid, are not necessarily sent for a bone scan. Another potential weakness is that we measured SUVmax. SUVmax has proven robust in oncology PET, although other parameters such as total lesion glycolosis (TLG) or SUVmean have been found more appropriate for some indications, including assessing response to treatment (Bai et al., 2013). We used SUVmax because of the issues regarding the reliability and reproducibility of VOI based measurement of myocardial uptake in normal hearts - it was one of our aims to establish a reference range for individuals without ATTR cardiac disease, individuals who by definition have only low grade myocardial uptake. Due to cardiac motion from contraction and positional variation related to breathing, the position of the heart on CT was not perfectly coregistered to the SPECT images, and on non-contrast low dose CT the myocardium is poorly visualised in normal individuals, hence defining a VOI which covered only myocardium was not practical in normal hearts. The advantage of SUVmax in this situation is that the location of the VOI is less important when a whole heart VOI is used, provided care is taken to exclude contamination from adjacent bone. Another approach would be to measure total uptake within a whole heart VOI, and this may be the most reliable way to assess disease progression/treatment response in HDP positive patients. However for the purposes of the current study we felt it was most appropriate to use the same method of determining the SUVs within all VOIs (including blood pool and bone) so that we could undertake subtraction and calculate ratios that mirrored Perugini grading if required. This allowed us to undertake an appropriate correction for blood pool. Ultimately these corrected myocardial uptake values proved sufficiently robust that they stood alone and no comparison to bone uptake was required. In addition the OSCGM reconstruction parameters we chose were those that produced a meaningful image visually, with a measurement in kBq/mL that approached the true value on initial phantom testing with relatively low image noise, reducing the chance that the SUVmax is significantly overestimating the uptake solely due to image noise.

Finally the relationship between the size of an object and the accuracy of quantitative SPECT/CT remains of theoretical concern (Bailey & Willowson, 2014). Most of the objects we measured were of a similar size across patients (such as aortic blood pool, vertebral body, rib) meaning that this potential error should not interfere with intersubject comparison. In regards to the myocardium itself this relationship is more complex and future modelling may produce techniques to confirm whether our measurements represent the true myocardial values in kBq/mL. However we have demonstrated that the current approach is clinically valid for single time point assessment. In addition the anatomy of the individual will most likely remain relatively constant over time, hence size is unlikely to confound comparison of serial studies.

## Conclusions

Quantitative SPECT/CT measurement of HDP myocardial uptake differentiates individuals with cardiac ATTR from the population not affected by this condition. This measurement of myocardial uptake of HDP stands alone, with no requirement for comparison to bone uptake. Furthermore this measurement produces a continuous variable which can be used to construct a reference interval for myocardial uptake for the population not affected by cardiac ATTR, against which single potentially affected individuals can be accurately differentiated. This technique uses a NIST traceable calibration source carrying with it the promise of developing multicentre reference intervals. Additional studies are warranted to determine if this measurement can be adapted to assess prognosis, progression of disease and response to treatment in cardiac ATTR.

## Abbreviations

AL: Amyloid light chain
ATTR: Amyloid transthyretin
HDP: 99mTechnetium-Hydroxymethylene diphosphonate
NIST: National Institute of Standards and Technology
VOI: Volume of interest
